# Predictors of Actual Turnover Among Part‐Time Homecare Nurses in Japan: A Longitudinal Study

**DOI:** 10.1002/jgf2.70121

**Published:** 2026-04-24

**Authors:** Maiko Noguchi‐Watanabe, Noriko Yamamoto‐Mitani, Hanako Numata, Asa Inagaki‐Asano

**Affiliations:** ^1^ Department of Gerontological Home Care and Long‐Term Care Nursing, Graduate School of Medicine The University of Tokyo Tokyo Japan

**Keywords:** communication, health services for the aged, homecare agencies, homecare services, longitudinal study, nursing personnel turnover

## Abstract

**Background:**

High turnover among homecare nurses leads to nursing shortages and a decline in care quality. In Japan, part‐time nurses are crucial to the workforce. However, their turnover predictors have not been extensively studied, despite the fact that they have higher turnover rates than full‐time nurses. This study aims to identify factors contributing to actual turnover among part‐time homecare nurses in Japan, providing insights for prevention.

**Methods:**

This longitudinal study analyzed data from 161 part‐time nurses who participated in a baseline survey in 2018 and a 2‐year follow‐up survey in 2020. The participants were selected from a baseline survey of 265 nurses. Data collected at baseline by homecare nurses included individual, work‐related, and workplace characteristics. The outcome was nurses' turnover (left or remained) 2 years later, as reported by nurse managers. The lengths of conversations with managers and colleagues were dichotomized into “short” and “long” using the lower 25th percentile as the cut‐off. Multivariate logistic regression analysis was conducted to identify predictors of turnover.

**Results:**

This study analyzed 161 part‐time nurses (mean age, 46.9 years). The turnover of nurses after 2 years was 32 (19.9%). Multivariate logistic regression analysis revealed that participants with short manager/long colleague conversations were less likely to experience turnover (odds ratio: 0.11; 95% confidence interval: 0.01–0.85) than those with short conversations with managers and colleagues.

**Conclusions:**

The results suggest that the combination of brief communication with managers and extended interactions with colleagues was associated with lower turnover among part‐time homecare nurses.

## 1 Introduction

Healthcare professionals provide support to clients in home healthcare settings, with homecare nurses serving a particularly vital role. However, excessive turnover contributes to nursing shortages, which in turn are associated with increased patient mortality, a higher incidence of adverse events, and a decline in the overall quality of care [1]. A shortage of homecare nurses can lead to difficulties in delivering essential nursing services and meeting patients' needs effectively [2]. Therefore, assuring a sufficient number of homecare nurses is essential.

The nursing workforce relies on full‐ and part‐time staff. In Japan, nursing staff are predominantly females (over 90%), and a significant proportion of these individuals opt for part‐time employment due to childcare and family responsibilities [3, 4]. The employment of full‐time staff alone is no longer sufficient, making consequently the hiring of part‐time nurses increasingly important. As a result, approximately 25% of nurses are employed part‐time. As the population in need of care continues to rise, the necessity of part‐time homecare nurses is increasing.

Part‐time nurses have different work situations compared with full‐time nurses: their job scope is narrower, emphasizing specific tasks, but features a higher degree of task‐based independence than that of full‐time nurses [5]. Part‐time nurses often work in positions with relatively low responsibility [5] and many have high perceived job insecurity [6]. Furthermore, part‐time work is associated with a higher odds of sickness absence (odds ratio: 1.09) [7] and lower physical function [8]. The limited opportunities for part‐time nurses to acquire new knowledge and skills, resulting from shorter working hours, have also been pointed out [5]. Thus, the part‐time work situation is inferred to be more complex and challenging than the full‐time environment.

Part‐time homecare nurses report a higher intention of turnover than full‐time nurses; the turnover rates for part‐time and full‐time nurses were reported as 26.3% and 15.6%, respectively [9]. The most common reason for part‐time nurse turnover is nurses' voluntarily leaving their jobs for employment at other healthcare organizations. In contrast, the most frequent reason for full‐time nurse turnover is health‐related issues; transferring to other organizations is not among the top reasons [10]. Therefore, part‐time nurse turnover may be reduced with some form of workplace support.

Various turnover predictors among homecare nurses, such as those presented by Ellenbecker and Cushman, have been examined in previous studies [11]. However, existing research has focused on examining the factors associated with turnover intentions [12, 13], and few studies have tracked actual turnover. A previous study reported that low job satisfaction, short tenure, and low intention to continue working are predictive factors of actual turnover among homecare nurses [14]. However, the study reporting these variables has been conducted in the United States under different healthcare and long‐term care systems and included both full‐ and part‐time nurses. Therefore, there is a need to identify predictors of actual turnover specifically for part‐time homecare nurses in the Japanese context.

Previous studies suggest that workplace communication is associated with turnover intention [15, 16]. Particularly in homecare settings, nurses often work alone, which can leave them feeling isolated from their organization [17]. Face‐to‐face communication at the office is a key opportunity for nurses to connect with managers and colleagues, and it is associated with turnover intention [18]. However, its direct effect on actual turnover has not been comprehensively examined. Analyzing these direct interactions may offer more practical intervention strategies for turnover prevention than relying on measures of general job satisfaction.

As the nature of the work differs between part‐time and full‐time nurses, identifying the predictors of turnover for part‐time nurses may enable the implementation of preventive measures. This study aimed to identify the predictors of turnover among part‐time homecare nurses working in Japan to provide methods for turnover prevention.

## 2 Methods

### 2.1 Design, Setting, and Participants

This study used longitudinal data of part‐time nurses who participated in a cross‐sectional study [18], which investigated their intentions to remain at the workplace and responded to a follow‐up survey at 2 years following the cross‐sectional study. We used these data to assess their turnover 2 years after the cross‐sectional study. At baseline, the study enrolled 265 part‐time nurses. Of these, 161 responded to the 2‐year follow‐up survey used here. The recruitment process has been detailed previously [18].

### 2.2 Data Collection

Data were collected in two waves: a baseline survey in 2018 and a follow‐up in 2020. To collect baseline data, questionnaires were sent to homecare managers, who distributed them to all nurses. Nurses completed the survey—providing information on individual and work‐related characteristics—and returned it directly to the researchers. Workplace characteristics, however, were reported by the homecare managers.

To ensure longitudinal data linkage, managers were requested by the researchers to maintain a table containing each nurse's unique ID for 2 years following the baseline survey. This allowed for the baseline questionnaires to be matched with the nurses' employment statuses (i.e., turnover) 2 years later. For the follow‐up survey, the researchers sent questionnaires—pre‐assigned with these IDs—to the organizations, and nurse managers provided the turnover statuses for the corresponding homecare nurses. Reminders were sent to both participating nurses and managers during both data collection periods.

### 2.3 Variables

#### 2.3.1 Outcome Variable

##### 2.3.1.1 Turnover

The outcome variable was the turnover of part‐time nurses within 2 years following the baseline survey. At the 2‐year data collection point, nurse managers reported on each homecare nurse's turnover status using four specific categories: left the job, remaining, transferred within the same organization, or on temporary leave. For the analysis, the outcome was dichotomized: (1) Turnover (left the job) and (2) Non‐Turnover (remaining, transferred, or on temporary leave).

#### 2.3.2 Independent Variables

Data on all independent variables were collected at baseline.

##### 2.3.2.1 Length of Conversation

We assessed the length of conversation based on type, degree, and frequency in one recent working day. Data on two types of nurse conversations were collected: a conversation between a nurse and their manager, and a conversation among nursing colleagues in a workplace. We excluded conversations from formal meetings, such as regular conferences and case meetings. Nurses reported the type and frequency of their conversations about their patients' conditions and nursing care on a single recent working day, along a time axis [18]. We did not specify the content of the conversations. To enable nurses to recall the time of their conversations from start to finish of the day, they were asked about the time they reached a client's home, moved to the next client's home, and attended an official conference.

##### 2.3.2.2 Individual Characteristics

The individual characteristics included sex, age, having a partner, having a child, being responsible for making a living, years of nursing and home care nursing experience, and years of working at the current workplace.

##### 2.3.2.3 Work‐Related Characteristics

The following work‐related characteristics were included: the number of working days per month, annual income, the total minutes of caregiving per day, minutes of moving per day, and the number of conferences or meetings per day.

Job satisfaction was measured using the Japanese version of the Home Healthcare Nurses' Job Satisfaction Scale [19], which consists of 30 items organized into seven subscales. Respondents rated each item using a Likert scale, ranging from “strongly disagree” to “strongly agree.” Scores ranged from 30 to 150, with higher scores indicating higher job satisfaction. The scale demonstrated adequate internal reliability, with acceptable Cronbach's alpha values for both the overall scale and its subscales.

##### 2.3.2.4 Workplace Characteristics

Variables regarding workplace characteristics included the number of nurses, the number of full‐time equivalent nurses, third‐party accreditation, unplanned visits, and the use of emergency medical resources such as emergency room visits and hospitalizations.

### 2.4 Statistical Analyses

First, we calculated descriptive statistics for the sample characteristics and determined the number of homecare nurses who experienced turnover. The distribution of each variable was examined. Univariate logistic regression analysis was used to investigate the relationship between each variable and turnover.

The length of the conversation did not show a normal distribution and was highly skewed. Therefore, it was treated as a categorical variable rather than a continuous one. The conversation was dichotomized into “short” conversation and “long” conversation for each interaction type: with the manager and colleagues. Participants who communicated less with both managers (< 20 min) and colleagues (< 40 min) were categorized under “short” communication. These cut‐off points were set at the lower 25th percentile for each conversation type, based on a previous study that identified these levels as significant predictors of the intention to remain [18]. This approach is consistent with common statistical practices, in which the quartile is used to define a high‐risk group in the absence of an established clinical threshold [20].

Before conducting multiple logistic regression analysis, we first checked for multicollinearity. Variables for the model were selected based on two criteria: statistical significance in the univariate logistic regression analysis and theoretical necessity (e.g., having a child, years of working at a current workplace, and minutes of caregiving per day). We used the forced entry method for the multiple logistic regression. However, the variables for the number of nurses, unplanned nurse visits per month, and emergency hospitalizations per 3 months were highly correlated (variance inflation factor [VIF] > 3). Consequently, we retained only the number of nurses, as it showed the strongest correlation with turnover, for inclusion in the final model.

Because the intraclass correlation coefficient (ICC) between homecare facilities was less than 0.1, a multi‐level analysis was deemed unnecessary. Referring to a previous study [14], the sample size was calculated by G*Power 3.1.9.2: estimated odds ratio = 2.5, *x* distribution = binominal, *α* = 0.05, power = 0.80. The minimum sample size was determined to be 151. All analyses were performed using Stata MP 15 (64‐bit) software (StataCorp LLC, College Station, TX, USA), with the level of statistical significance set at *p* < 0.05.

### 2.5 Ethical Approval

This study was approved by the University of Tokyo Research Ethics Service Committee (No. 11875). Participants were provided the questionnaire and a research summary describing the purpose and outline of the 2‐year study. We emphasized that participation in the study was voluntary. Informed consent was obtained from all individual participants included in the study.

## 3 Results

Of 265 part‐time nurses who returned questionnaires at baseline, we obtained the turnover statuses of 161 homecare nurses 2 years later. In total, 161 valid responses were analyzed (valid response rate: 60.8%). The individual characteristics of participants who were followed up at 2 years and those who were not followed up were not significantly different (Table S1).

Table 1 shows the participant characteristics. Almost all participants (98%) were females, and the mean age was 46.9 years. Approximately 90% had a child or children, and the mean number of years at their current workplace was 6.1 years. In the work‐related characteristics, the mean length of conversation per day was 85.8 min. The mean length of conversation was 28.15 min with managers and 57.24 min with colleagues. The combinations of conversation lengths were as follows: (1) short with both managers and colleagues (*n* = 22, 13.7%), (2) short with managers and long with colleagues (*n* = 31, 19.3%), (3) long with managers and short with colleagues (*n* = 24, 14.9%), and (4) long with both managers and colleagues (*n* = 83, 51.6%). At baseline, 8.7% participants had turnover intention. Two years later, 32 participants experienced turnover (19.9%). The reason for turnover was classified as ‘other’ or ‘unknown’ in over 50% of the turnover cases. Excluding these categories, childbirth/children (*n* = 4, 12.2%) was identified as the most common reason for turnover.

**TABLE 1:** Baseline participant characteristics and turnover.

		Part‐time nurse (*n* = 161)			
		Mean	SD	*n*	%
*Baseline*					
Individual characteristics					
Sex	Female			159	98.8
Age (years)		46.9	8.0		
Having partner				141	87.6
Child	Yes			149	92.5
Responsible for making a living	Yes			20	12.4
Nursing experience (years)		20.1	7.7		
Homecare nursing experience (years)		7.8	6.1		
Working at a current workplace (years)		6.1	5.2		
Work‐related characteristics					
Working days per month		16.6	4.3		
Annal income ¥3,000,000 and above				38	23.6
Less than ¥3,000,000				122	75.6
Caregiving per day (minutes)		220.4	59.2		
Moving per day (minutes)		92.5	35.1		
Conference/meeting (minutes)		28.3	30.8		
Job satisfaction		105.5	0.8		
Turnover intention				14	8.7
Workplace characteristics					
Actual number of nurses		9.7	5.7		
Full‐time equivalent nurse		8.6	4.1		
Accreditation by third‐party	Yes			130	80.7
	No			21	13.0
Unplanned homecare nurse visits (per month)	56.1	42.5			
Emergency room visit (per 3 months)		4.3	3.7		
Emergency hospitalization (per 3 months)		9.8	8.9		
Length of conversation					
With managers and colleagues (min/day)		85.82	56.0		
With managers (min/day)		28.15	28.3		
With colleagues (min/day)		57.24	39.0		
The combinations of conversation length pattern (Manager/Colleagues)^a^					
Short/Short				22	13.7
Short/Long				31	19.3
Long/Short				24	14.9
Long/Long				83	51.6
*Turnover*				32	19.9
Relocation				1	3.0
Illness				3	9.1
Childbirth/childcare				4	12.2
Other				18	54.5
Unknown				6	18.1
Stay				129	80.1

Abbreviation: SD, standard deviation.

^a^ Conversations at a workplace consisted of conversations with managers and with colleagues. The length of conversation with managers was divided into short (< 20 min/day) and long (≥ 20 min/day) groups. The length of conversation with colleagues was divided into short (< 40 min/day) and long (≥ 40 min/day) groups.

Table 2 shows the incidence of nurse turnover relative to combinations of conversation length with managers and colleagues. After 2 years, turnover was observed in 32 nurses (19.9%). An examination of the four conversation length patterns revealed that the highest turnover rate (36.4%) occurred among nurses who reported short conversations with both managers and colleagues. In contrast, the other patterns showed a lower turnover incidence, ranging from 12% to 19%.

**TABLE 2:** Combinations of conversation length patterns and actual turnover at 2 years.

	Actual turnover				Total	Incidence rate
	Turnover		Stay			
The combinations of conversation length pattern (Manager/Colleagues)^a^						
Short/Short	8	(36.4)	14	(63.6)	22	0.36
Short/Long	5	(16.1)	26	(83.9)	31	0.16
Long/Short	3	(12.5)	21	(87.5)	24	0.13
Long/Long	16	(19.0)	68	(81.0)	84	0.19
Total	32	(19.9)	129	(80.1)	161	0.20

*Note:* Incidence rate = Number of nurses who actually left/total number of nurses.

^a^ Conversations at a workplace consisted of conversations with managers and with colleagues. The length of conversation with managers was divided into short (< 20 min/day) and long (≥ 20 min/day) groups. The length of conversation with colleagues was divided into short (< 40 min/day) and long (≥ 40 min/day) groups.

The results of a logistic regression analysis are shown in Table 3. In the adjusted model, the short conversation with the manager/long conversation with colleagues combination was significantly associated with a lower likelihood of turnover (adjusted odds ratio [AOR]: 0.11, 95% confidence interval [CI]: 0.001–0.85, *p* = 0.035). Conversely, job satisfaction (AOR: 0.94, 95% CI: 0.87–1.01, *p* = 0.089) and turnover intention at baseline (AOR: 2.03, 95% CI: 0.48–8.39, *p* = 0.329) were not identified as significant factors for turnover.

**TABLE 3:** Association between the length of conversation and homecare nurses' turnover.

	COR	95% CI	*p*	AOR	95% CI	*p*
Length of conversation						
Minutes per day						
With managers and colleagues	0.44	0.10–1.95	0.28			
With managers	0.99	0.97–1.01	0.18			
With colleagues	0.99	0.98–1.00	0.33			
Length of conversation (short/long)^a^						
Short with managers and colleagues [ref. long]	0.44	0.10–1.95	0.28			
Short with managers [ref. long]	1.10	0.65–1.89	0.72			
Short with colleagues [ref. long]	1.16	0.68–1.98	0.59			
The combinations of conversation length pattern (Manager/Colleagues)						
Short/Short	ref			ref		
Short/Long	0.34	0.09–1.22	0.10	0.11	0.01–0.85	0.04
Long/Short	0.03	0.06–1.11	0.07	0.23	0.03–1.56	0.13
Long/Long	0.41	0.15–1.15	0.09	0.37	0.10–1.30	0.12
Individual characteristics						
Sex	4.00	0.24–65.7	0.34			
Age	0.99	0.95–1.04	0.77			
Having partner	0.41	0.13–1.14	0.11			
Child	0.20	0.95–1.09	0.07	0.20	0.03–1.17	0.07
Responsible for making a living	0.17	0.12–0.89	0.03	0.28	0.07–1.12	0.07
Nursing experience (years)	1.01	0.96–1.06	0.76			
Homecare nursing experience (years)	0.98	0.92–1.05	0.67			
Working at a current workplace (years)	0.96	0.88–1.04	0.27	1.01	0.92–1.11	0.81
Work‐related characteristics						
Working days per month	1.03	0.94–1.13	0.49			
Annual income ¥3,000,000 and above	1.03	0.40–2.53	0.94			
Less than ¥3,000,000	1.00					
Care giving per day (minutes)	1.00	0.99–1.01	0.16	0.99	0.98–1.01	0.19
Moving per day (minutes)	1.00	0.99–1.02	0.34			
Conference/meeting (minutes)	1.00	0.98–1.01	0.70			
Job satisfaction	0.94	0.89–0.99	0.03	0.94	0.87–1.01	0.09
Turnover intention	1.95	0.63–6.02	0.24	2.03	0.48–8.39	0.33
Workplace characteristics						
Actual number of nurses	1.00	0.94–1.07	0.94			
Full‐time equivalent nurse	0.98	0.88–1.08	0.66			
Accreditation by third‐party	1.26	0.42–3.76	0.68			
Unplanned homecare nurse visits (per month)	1.00	0.99–1.01	0.71			
Emergency room visit (per 3 months)	0.85	0.73–0.98	0.03	0.85	0.72–1.06	0.08
Emergency hospitalization (per 3 months)	0.99	0.95–1.04	0.72			

*Note:* Logistic regression analysis.

Abbreviations: AOR, adjusted odds ratio; CI, confidence interval; COR, crude odds ratio.

^a^ Conversations at a workplace consisted of conversations with managers and with colleagues. The length of conversation with managers was divided into short (< 20 min/day) and long (≥ 20 min/day) groups. The length of conversation with colleagues was divided into short (< 40 min/day) and long (≥ 40 min/day) groups.

## 4 Discussion

This study investigated the 2‐year turnover rate among part‐time homecare nurses and identified its predictors. Notably, while many previous studies in nursing management have focused on turnover intention, this study provides empirical evidence of a direct link between face‐to‐face conversation and actual turnover. Our results showed that the combination of short manager conversations and long colleague conversations is significantly associated with a decreased likelihood of turnover. These findings provide valuable insight for nursing managers and other stakeholders: ensuring time for conversations with colleagues may help prevent early part‐time homecare nurse turnover.

The 2‐year turnover rate observed in our study (19.9%) was notably lower than the 26.3% 1‐year turnover rate reported in a previous study [9]. Foremost, the larger average workplace size in the present study (8.6 FTE nurses) was likely associated with the lower turnover incidence. This finding aligns with prior research [21], which indicates that larger workplaces typically exhibit lower turnover rates. Secondly, the timing of data collection may be a crucial factor. Our study period (2018–2020) coincided with the beginning of the COVID‐19 pandemic, whereas the comparison study [9] was conducted in 2024, after the healthcare system had adapted, and nurse turnover intentions were reported to be higher than pre‐pandemic levels [22]. This temporal difference likely impacted the observed turnover rates.

A long conversation with colleagues combined with short conversations with managers was associated with a lower likelihood of turnover. Workplace stress is a common reason for nurse turnover [23]. Part‐time nurses often perceive themselves as outsiders, which increases stress, and they need psychological support [24]. Sufficient communication among colleagues at the workplace may offer crucial psychological support, helping part‐time nurses to avoid feeling marginalized. This suggests that ensuring sufficient face‐to‐face interaction can mitigate workplace isolation, which this study identified as a key factor associated with actual turnover. Thus, facilitating opportunities for nurses to engage in conversations with colleagues may be an effective strategy for employers to reduce turnover among part‐time nurses.

Specifically focusing on the manager conversation length, the combination of a short conversation with managers and a long conversation with colleagues significantly predicted lower turnover. This may reflect the time constraints faced by part‐time nurses [5], suggesting that intense verbal communication with managers delivered in a short timeframe may be highly effective.

Because a manager's nonverbal communication is known to relate to staff motivation [25], integrating nonverbal cues and using communication tools should be considered as a part of future strategies to reduce staff turnover. Another possible explanation involves the influence of management leadership style on staff stress [26]. Since the appropriate leadership style depends on the organization's characteristics and maturity [27], extended conversations with managers may increase stress among part‐time nurses if the leadership approach is mismatched. Future research should examine the role of leadership styles in predicting homecare nurse turnover.

Job satisfaction and turnover intention were not identified as significant predictors of turnover. Although job satisfaction is significantly related to turnover intention [28, 29], the relationship between job satisfaction and actually leaving varies among studies [14, 30]. Given that turnover intention is known to be volatile and subject to change [30], its measurement solely at baseline in this study likely limited its ability to predict actual turnover behavior during the 2‐year follow‐up period. This discrepancy suggests the potential importance of focusing on objective communication factors and actual turnover outcomes, complementing previous findings that relied solely on turnover intention.

Considering the scope of this study, which exclusively utilized face‐to‐face conversation length, the results underscore the importance of in‐person interaction in this context. The length of exclusively face‐to‐face conversation significantly predicted actual turnover among part‐time homecare nurses. This finding suggests that the high synchronicity inherent in face‐to‐face communication [31]—meaning the exchange of information occurs instantaneously—is likely essential for managing work demands and maintaining psychosocial balance in roles that require immediate, critical judgment, such as responding to sudden changes in patient condition during home visits [32]. Future research should, therefore, investigate how asynchronous communication tools, such as email or bulletin boards, affect turnover behavior, especially among part‐time nurses with shorter working hours.

This study has some limitations. First, since some participants were lost to follow‐up over the 2‐year period, there is a possibility that the turnover rate was underestimated. This missing data may have led to reduced statistical power, thereby potentially concealing true predictive factors for actual turnover. Future studies necessitate a longer follow‐up period with rigorous cohort tracking, such as implementing regular check‐ins and collecting multiple forms of contact information at baseline. Second, predictive factors may also differ depending on the reason for turnover. However, owing to the limited sample size in this study, all reasons for leaving were analyzed together. In future studies, it will be necessary to increase the number of participants with the intent to analyze turnover reasons separately and to examine the predictors of leaving for each specific cause. Third, there may be a bias in the geographical representation of the participants. This study targeted homecare nurses within only two specific geographical areas, which limits the generalizability of the results to the entire country. However, since the study targeted both rural and urban areas in Japan, a certain degree of generalizability may still be ensured. In the future, a study utilizing a highly representative dataset of participants selected by random sampling from across the country is required. Finally, this study focused solely on the length of conversations, precluding an analysis of the impact of conversation content on turnover. For instance, the impact of gossip versus constructive discussions about patient care on homecare nurses' turnover may differ significantly. Therefore, future research should incorporate conversation content into its analysis.

In conclusion, this study investigated the predictors of actual turnover among part‐time homecare nurses in Japan. The 2‐year turnover rate was 19.9%, and the combination of short conversations with managers and long conversations with colleagues significantly predicted lower turnover among part‐time homecare nurses. Workplace managers and supervisors of homecare nurses may help prevent part‐time homecare nurses from immature turnover by providing support that allows them to spend more time in conversation with their colleagues. Future research must expand upon these findings by incorporating the content of conversations and management leadership styles to develop more comprehensive retention strategies.

## Author Contributions

**Hanako Numata:** writing – review and editing, methodology, supervision, resources. **Noriko Yamamoto‐Mitani:** supervision, conceptualization, writing – review and editing. **Asa Inagaki‐Asano:** methodology, writing – review and editing. **Maiko Noguchi‐Watanabe:** conceptualization, investigation, funding acquisition, writing – original draft, methodology, validation, visualization, writing – review and editing, formal analysis, project administration, data curation, software.

## Funding

This work was supported by the JSPS KAKENHI (Grant Numbers: 16K20970, 23K27912).

## Consent

Informed consent was obtained from all individual participants included in the study.

## Conflicts of Interest

The authors declare no conflicts of interest.

## Supporting information


**Table S1:** Baseline characteristics between participants who were followed up and those who dropped out.

## Data Availability

The data is not publicly available due to privacy or ethical restrictions but can be provided by the corresponding author upon reasonable request.
