# Supplementary material for: Predictors of Actual Turnover Among Part‐Time Homecare Nurses in Japan: A Longitudinal Study
Source: J Gen Fam Med. 2026 Apr 24;27(3):e70121. doi: 10.1002/jgf2.70121 (PMC13109612; doi:10.1002/jgf2.70121)
Supplement: Supplementary file 1 — Table S1: Baseline characteristics between participants who were followed up and those who dropped out. [file JGF2-27-e70121-s001.docx]

**Supplemental Table 1. Baseline characteristics between participants who were followed up and those who dropped out**

|  |  | Follow-up (n=161) | Drop out (n=104) | *p* |
| --- | --- | --- | --- | --- |
| **Individual characteristics** |  |  |  |  |
| Sex | Female | 159 (98.8) | 104 (100.0) | 0.249 |
| Age (years) |  | 46.8±8.0 | 47.5±7.8 | 0.317 |
| Having partner |  | 141 (87.6) | 93 (89.4) | 0.785 |
| Child | Yes | 149 (92.5) | 94 (90.4) | 0.391 |
| Responsible for making a living | Yes | 20 (12.4) | 10 (9.6) | 0.434 |
| Nursing experience (years) |  | 20.1±7.7 | 22.1±8.8 | 0.052 |
| Home care nursing experience (years) |  | 7.8±6.1 | 7.8±5.9 | 0.923 |
| Working at a current workplace (years) | | 6.1±5.2 | 6.7±5.7 | 0.454 |
| **Work-related characteristics** |  |  |  |  |
| Working days per month |  | 16.6±4.3 | 16.6±4.6 | 0.861 |
| Annal income ¥3,000,000 and above |  | 38 (23.9) | 31(29.8) | 0.287 |
| Care giving per day (minutes) |  | 220.4±59.2 | 251.9±75.8 | <0.001 |
| Moving per day (minutes) |  | 92.5±35.1 | 96.8±41.1 | 0.337 |
| Conference/meeting (minutes) |  | 28.3±30.8 | 33.1±28.7 | 0.04 |
| Length of conversation (minutes per day) |  |  |  |  |
| with a manager and colleagues |  | 85.8±56.0 | 85.9±61.0 | 0.628 |
| with a manager |  | 28.2±28.3 | 29.8±31.9 | 0.811 |
| with colleagues |  | 57.2±39.0 | 55.3±40.9 | 0.377 |
| Combination of the length of conversation |  |  |  | 0.743 |
| Short conversation (manager) × short conversation (colleagues) | | 22 (13.7) | 17 (10.6) |  |
| Short conversation (manager) × long conversation (colleagues) | | 31 (19.3) | 18 (11.2) |  |
| Long conversation (manager) × short conversation (colleagues) | | 24 (14.9) | 20 (12.4) |  |
| Long conversation (manager) × long conversation (colleagues) | | 83 (51.6) | 51 (31.7) |  |
| Job Satisfaction | | 105.5±0.8 | 105.2±1.0 | 0.792 |
| Turnover intention | | 14 (8.7) | 12 (7.5) | 0.479 |
| **Workplace characteristics** |  |  |  |  |
| Actual number of nurses |  | 9.7±5.7 | 11.7±8.6 | 0.021 |
| Full-time equivalent nurse | | 8.6±4.1 | 8.9±5.8 | 0.604 |
| Accreditation by third-party | Yes | 131 (81.4) | 78 (75.0) | 0.011 |
| Unplanned homecare nurse visits (per month) |  | 56.1±42.5 | 73.6±68.2 | 0.103 |
| Emergency room visit (per 3 months) | | 4.3±3.7 | 2.6±1.2 | 0.018 |
| Emergency hospitalization (per 3 months) |  | 9.8±8.9 | 3.7±3.2 | 0.007 |

*Note.* Mean ± Standard Deviation, or n (%), Chi-square test, or t-test.
